# Refining Trait Resilience: Identifying Engineering, Ecological, and Adaptive Facets from Extant Measures of Resilience

**DOI:** 10.1371/journal.pone.0131826

**Published:** 2015-07-01

**Authors:** John Maltby, Liz Day, Sophie Hall

**Affiliations:** 1 College of Medicine, Biological Sciences, and Psychology, University of Leicester, Leicester, United Kingdom; 2 Department of Psychology, Sociology and Politics, Sheffield Hallam University, Sheffield, United Kingdom; 3 School of Life Sciences, University of Lincoln, Lincoln, United Kingdom; Defence Science & Technology Organisation, AUSTRALIA

## Abstract

The current paper presents a new measure of trait resilience derived from three common mechanisms identified in ecological theory: Engineering, Ecological and Adaptive (EEA) resilience. Exploratory and confirmatory factor analyses of five existing resilience scales suggest that the three trait resilience facets emerge, and can be reduced to a 12-item scale. The conceptualization and value of EEA resilience within the wider trait and well-being psychology is illustrated in terms of differing relationships with adaptive expressions of the traits of the five-factor personality model and the contribution to well-being after controlling for personality and coping, or over time. The current findings suggest that EEA resilience is a useful and parsimonious model and measure of trait resilience that can readily be placed within wider trait psychology and that is found to contribute to individual well-being.

## Introduction

In the wider literature, studies have tended to operationalize psychological resilience in one of two ways: either (1) as part of a process or state (i.e. the buffering approach [1–4]), or (2) as a trait (e.g. [5–6]). These are not necessarily competing approaches, both having much to commend them. The buffering approach examines resilience on a bipolar dimension, as the opposite of risk, examining whether specific psychological characteristics or processes interact with particular negative events as resilience buffers to reduce their impact [1–4]. In comparison, the assessment of trait resilience examines how individuals approach and react in general to events that they experience to be negative, and considers their ability to recover from these negative events. Whilst the buffering approach is now well-operationalized [1], there is much less consensus about what constitutes trait resilience.

The lack of clarity concerning what constitutes trait resilience is highlighted in Windle, Bennett, and Noyes’ [7] methodological review of fifteen original measures and four refinements of existing self-report measures of resilience. The measures were drawn from a wide variety of theoretical and empirical contexts, including hardiness, adaptive coping and protective factors, resources, perseverance, impulse control, self-esteem and social interaction. Windle et al. noted that whilst each measure provides well-operationalized and valuable assessments of specific aspects of resilience, there is no "gold standard" (p. 1) for measuring resilience. Similarly, Pangello, Zibarrass, Lewis, and Flaxman [8] assessed seventeen resilience measures (with some overlap with Windle et al.'s inclusion criteria) using an interactionist theoretical framework and indentified nine themes: adaptability, self-efficacy, active coping, positive emotions, mastery, hardiness, supportive relationships, structured environment, and conceptual adequacy. Overall, Pangello et al. [8] point out an inconsistency across the literature in terms of the "definition and operationalization of resilience that warrant further consideration" (p. 1).

The current study aimed to develop a more parsimonious and therefore valuable approach to assessing trait resilience, by exploring and consolidating the variety of theoretical and empirical approaches currently used to assess it. To begin this process we adopt an approach proposed and developed by Holling and colleagues that combines systems theory and ecology to describe resilience across a number of ecological systems [9–13]. Within this approach there are three related broad systems surrounding resilience: engineering resilience, ecological resilience, and adaptive capacity. Engineering resilience, so named because it is often referred to in engineering and physical science systems, is the ability (in terms of speed or status) of any system to return to, or recover, an equilibrium following any disturbance [9, 11, 13]. Ecological resilience is the ability of a system to absorb or resist a perturbation before realigning the key mechanisms of the system, and to maintain its stable state, in terms of function, purpose, structure, or identity [11, 14]. Ecological resilience focuses on the magnitude of disturbance that can be absorbed or resisted by the system, while the system simultaneously monitors and reorganizes the processes that govern the system's behaviour so as to accommodate or resist the disturbance [13]. Adaptive capacity is the ability of an ecosystem to manage and accommodate change, and to adapt. A key aspect to adaptive capacity is that systems are resilient due to their ability to persistently resist disturbance by continually varying their key functions and processes [9, 12, 15–16].

These systems of trait resilience are evidenced within the wider academic literature across a range of biological, environmental, and socially resilient systems. Engineering resilience (through the ability to rebound, heal, return to an equilibrium state, or bounce back) has been portrayed as resilience in the explanation of equilibrium states in water column disturbances in microbiology, dynamic restoration in coastal dunes, and the restoration of critical ecosystem services [17–19]. Ecological resilience (through the ability to be robust, permanent or persistent) has been described as resilience in the explanation of resistant bacteria responses to water column disturbances in microbiology, the strong responses of receptors and mechanisms in biology, stability among complex networks within financial markets, the permanence of farm systems in ecology, and the permanence of non-democratic political systems [17, 20–25]. Adaptive capacity (through the ability to restructure, transform, or materialize) has been depicted as resilience in the explanation of genetic diversity within tolerant genotypes in toxicology, the activities of receptors in biology, the ability to respond effectively to extreme events in social ecology, and democratic systems in politics [20–21, 26–28]. Finally, though not formulated within any single theoretical or measurement model of psychological trait resilience, these three themes feature repeatedly in the psychological literature. Engineering resilience has been recognized in the psychological literature as an individual’s capacity to rebound or 'bounce back' to their original state following difficult experiences [29–33]. Ecological resilience has been recognized in the psychological literature as the capacity to be robust, demonstrating confidence in one's strengths and abilities, and being stoical, resourceful, and determined as one navigates through key domains across and within one’s life [29, 34–37]. Adaptive capacity has been recognized in the psychological literature as the ability to adapt well, adjust, be flexible, change, innovate, modify, and respond well to disturbances [29, 38–40].

The first consideration of the current research is to explore the proposal that, within human behavior, there are three main domains to trait resilience: Engineering resilience, Ecological resilience and Adaptive capacity (henceforth collectively referred to in this paper as the EEA resilience model). The paper achieves this by first considering the relationship between items of existing measures of trait resilience. Kline [41] has argued that a central function of psychometric techniques, such as factor analysis, is that they can provide evidence of the validity of proposed traits by identifying latent constructs underpinning a number of items among the population at any single time. Given the proposed relevance of engineering, ecological, and adaptive resilience to ecological systems across a number of system domains, it is argued that, if they are relevant to psychological theory, these three systems should reveal themselves as traits within exploratory factor analytic approaches [41]. Therefore, the validity of the EEA trait resilience concepts could be tested by examining whether the EEA system emerges when considering the structure of items contained within existing measures of psychological resilience, regardless of whether those existing measures directly measure the EEA system.

To test whether the EEA system emerges in a factor analysis of existing measures of resilience, we chose five established measures of self-report psychological resilience that have been posited to assess resilience traits (albeit described as traits, characteristics, or personal qualities): the *Ego Resiliency Scale* (ER89; [42]), the *Hardiness Scale* [5], the *Psychological Resilience Scale* [6], the *Connor-Davidson Resilience Scale* (CD-RISC; [43]) and the *Brief Resilience Scale* [30]. When these five measures are split into their respective subscales they measure nine aspects of resilience. The ER89 [42] assesses the capacity of the individual to demonstrate control, in terms of impulses or inhibition, in response to environmental demands, in order to safeguard or augment the ego equilibrium. The Hardiness Scale [5] has its theoretical origins in existential personality theory [44], with a wider discussion of whether hardiness factors are dispositions [44], attitudes [45–46], or a broad personality style encompassing cognitive, emotional, and behavioral traits [47]. Hardiness comprises three general dimensions: (1) commitment (a belief that life is meaningful), (2) control (self-efficacy in life), and (3) challenge (ability to, and enjoyment of, change) [44, 48]. The Psychological Resilience Scale [6] measures a "resilience core", defined by five characteristics (purposeful life, perseverance, self-reliance, equanimity, and existential aloneness) reflecting an individual's overall physical and mental health resilience across the life trajectory. The CD-RISC [43] was developed within a clinical treatment context that viewed resilience as coming from four sources: (1) control, commitment, and change hardiness constructs [44], (2) goal-orientated strategies, encompassing concepts such as confidence, an adaptive nature, problem solving and coping [49], (3) the constructs of patience and the ability to endure stress [50], and (4) faith and good fortune [43, 51]. Finally, the Brief Resilience Scale [30] comprises a single component designed to measure resilience as the ability to recover from adverse situations.

The first four scales were chosen because of their contribution to the psychological literature, being the most cited scales. The total number of times these four scales have been cited exceeds 1500, with the minimum number of citations 255 for the Hardiness Scale [52]. The Brief Resilience Scale has been cited to a lesser degree (63 times) [52], likely due to the relative recency of its publication. However, we included this scale because it has a good citation rate since publication, and because it clearly measures a construct relating to engineering resilience. In Windle et al.'s [7] quality assessment (in terms of overall reliability, validity, and clinical and measurement sensitivity) of psychometric scales of resilience, four of the five scales (the exception being the Hardiness Scale) received a score of either 6 or 7 (with a top score of 7). However, we retained the Hardiness Scale given its early prominence in the resilience literature and its applicability to assessing a range of resilience traits via psychological, personality-based, and cognitive hardiness across a large number of items [5, 44–46].

None of the aforementioned five available measures of psychological resilience explicitly contain scales or subscales that will allow us to consider all aspects of the EEA trait resilience. However, concepts similar to those abilities described in the EEA model are considered in the descriptions or items of these measures: Engineering resilience is reflected in the Brief Resilience Scale [30], in which the ability to recover and return to one's state, in the context of speed, is measured across a number of items (e.g. "Does not take a long time to recover"). Ecological resilience is considered in the ER89 [42] where attempts to maintain or safeguard the ego equilibrium are generally examined (e.g. "*Strong* personality"), in the Psychological Resilience Scale [6] where traits such as perseverance and equanimity are assessed (e.g. "When things look hopeless, I don't give up"), and in the CD-RISC [43] where endurance and the ability to maintain and manage process is a consideration (e.g. "Under pressure, focuses and thinks clearly"). Adaptive resilience is considered in the Hardiness Scale [5] where ability to, and enjoyment of, change are assessed (e.g. "Changes in routine are interesting") and in the ER89 [42] where traits around being adaptive are considered (e.g. "Likes new and different things"). However, the scales also contain items that refer to a number of constructs (e.g. self-reliance [5], existential aloneness [6], patience, faith, and good fortune [43]) that are not directly defined within the model of EEA trait resilience but do appear within other models of resilience. This provides necessary "controls" that ensure a proper test of whether EEA trait resilience emerges as latent factors within existing measures of resilience. Therefore, by identifying the latent factors from these scale items we will test the proposition that the EEA trait resilience systems should emerge as latent traits among a collection of items posited to, and having an evidential basis for, assessing trait resilience.

A second consideration of the current research is the importance of EEA trait resilience in terms of other domains within psychology. By examining the differing relationships between EEA trait resilience and personality and coping, we hope to augment some of the theoretical and empirical underpinnings of each of the EEA constructs. Research suggests that measures of resilience are related to the adaptive traits found within the five-factor model of personality, via a positive correlation with extraversion, agreeableness, openness, and conscientiousness, and a negative association with neuroticism [53–55]. Also, findings suggest that measures of resilience are positively associated with adaptive coping traits such as problem-focused coping, positive appraisals, and emotional regulation [56–57], not least because resilience is identified as a characteristic of positive coping [58]. Therefore, it is expected that the facets of EEA trait resilience should fit positively into the "adaptive landscape" [59, p. 471] of positive expressions of personality and coping traits [59, 60]. Additionally, given the emphasis on well-being within the resilience literature [61, 62], we consider two ways in which EEA trait resilience is related to well-being. First, we examine the usefulness of the EEA model for predicting well-being, over and above the existing models of personality and coping, as neuroticism (negatively) and extraversion (positively) are strongly related to well-being [63–64], and higher levels of adaptive coping [65] are reported to have a positive association with well-being. Second, we examine the extent to which trait resilience contributes to changes in well-being over time, thereby providing some predictive value for the EEA model.

In summary, we tested the proposed EEA model of trait resilience, comprising the traits of engineering, ecological, and adaptive resilience. The approach we adopted was to consider the measurement of the three main aspects of trait resilience by (1) exploring the psychometric properties of the items of five existing measures of trait resilience (Study 1), and considering (2) the relationship of EEA trait resilience to trait psychology variables of personality and coping and (3) the value of EEA trait resilience in predicting well-being after controlling for personality and coping, and over time (Study 2).

## Study 1

The aim of Study 1 was to consider the measurement of adaptive, impervious, and engineering resilience by exploring the psychometric properties of the items of five existing measures of trait resilience.

### Materials and Method

#### Sample

Two samples of data were collected. Sample 1 was used for an exploratory factor analysis (EFA) and Sample 2 for a confirmatory factor analysis (CFA).

The first sample comprised 622 respondents (90 men, 532 women), aged 18 to 45 years (*M* = 20.20 years, *SD* = 3.0), who were either undergraduates or postgraduates enrolled on psychology courses at United Kingdom universities, sampled over a three-year period. Three hundred and ninety one respondents reported that they were of a white ethnicity, 108 respondents reported themselves as Asian, 61 as Black, 27 as being of mixed race, 25 as falling into the "other" category, and 4 as Middle Eastern. Six respondents did not reveal their ethnicity. The sample comprised participants in a university experiment participation scheme, whereby students were given the choice to take part in experiments in return for being able to recruit participants for their own research projects in their final year. The study was advertised and volunteers signed up and completed the study online via an electronic survey system. If participants withdrew from a single study or multiple studies under the scheme they did not jeopardize the reward (recruiting participants for their own research projects).

The second sample comprised 168 older adults (70 men and 98 women) aged 18 to 70 years (*M* = 35.91 years, *SD* = 8.7), drawn from the wider general population. 123 participants reported themselves as White, 25 as Asian, 18 as Black, and 2 as being of mixed race. The respondents were drawn from a number of occupations; the majority reported being in the service industry (15 respondents) or education (14 respondents). The recruitment combined opportunistic and snowball sampling, with a number of individuals being contacted in the first instance to complete the questionnaire via major social networking sites and then being asked to pass on details of the study to acquaintances on these sites. Twenty-seven responses were removed because the respondents were in the same occupational group (e.g. students) as the participants in the first sample as we were unable to determine whether they had already been included in that sample.

#### Materials

The respondents in Sample 1 were asked to complete the following five resilience scales:

The Ego Resiliency Scale (ER89 [42]). This 14-item scale assesses an individual’s ability to adapt to a stressful experience and return to their own individual characteristics afterwards. Responses are scored on a seven-point scale from 1 (*Does not apply at all*) to 7 (*Applies very strongly*). The internal reliability of ER89 has been reported to be α = .76 [42].

The Hardiness Scale [5]. This scale comprises 45 items designed to measure dispositional resilience, presented as three factors: commitment, control, and challenge. Responses are scored on a four-point scale ranging from 0 (*Not at all true*) to 3 (*Completely true*). The subscales demonstrate good internal consistency (α = .90–.93) and predictive validity for psychological and physical health [5].

The Psychological Resilience Scale [3]. This 25-item scale measures resilience via the capacity to withstand stress and create meaning from challenges. Responses are scored on a seven-point scale from 1 (*Disagree*) to 7 (*Agree*). The scale demonstrates good internal consistency (α = .72 to .94) and its construct validity is evidenced by expected correlations between scores on the scale, and stress, anxiety, and health-promoting activities [66].

The Connor-Davidson Resilience Scale (CD-RISC [43]). The CD-RISC is a 25-item measure of a series of trait characteristics that are thought to exemplify resilience via personal competence, strengthening effects of stress, secure relationships, control, and spiritual influences. Responses are scored on a five-point scale ranging from 0 (*Not at all true*) to 4 (*True nearly all of the time*). The scale has demonstrated good internal consistency (α = .89), test-retest reliability (*r* = .87), and appropriate convergent validity via its expected relationships with other measures of hardiness and stress [43].

The Brief Resilience Scale [30]. This six-item scale measures an individual’s ability to "bounce back" from stressful situations or adversity. Items are scored on a five-point response scale from 1 (*Strongly disagree*) to 5 (*Strongly agree*). The scale has demonstrated good internal consistency (α = .80 –.91), test-retest reliability (*r* = .62 –.69) and appropriate correlations with a number of psychological (e.g. affect, stress, optimism) and health well-being (e.g. fatigue) variables [30].

Participants in Sample 2 were asked to complete 12 items that were suggested by the EFA as being appropriate for measuring EEA trait resilience. To standardize the rating scales, the response format for all items was changed to a four-point scale: 1 (*Strongly disagree*), 2 (*Disagree*), 3 (*Agree*), and 4 (*Strongly agree*).

#### Procedure

For all surveys, the electronic survey system was set up in such a way that the respondents had to answer all the questions. It was not possible to record how many participants simply did not complete the online survey due to the design of the software, though participants were provided with an easy way to withdraw from the study by simply ceasing to fill out the scale online. In Sample 1, the number of respondents who successfully completed the survey represented the large majority of participants (over 84%) who took part in the university experiment participation scheme over that time. Therefore, given the completion rate, there does not seem to be any strong suggestion of particular attrition or reactance effects due to the use of this forced completion method. Furthermore, the software used with this sample recorded how much time each respondent spent on the survey. 98% of the respondents spent 22 minutes or more completing the survey (with no respondent spending less than 19 minutes), suggesting that the forced completion of the survey did not necessarily lead to people rushing to complete the survey. Among the undergraduate and postgraduate students, the software allowed the order of the administration of the scales to be randomized. The randomization of the scales was not necessary with the older adult sample as only one scale was administered.

#### Ethical Consent

The study procedure received ethical approval from the University of Leicester’s School of Psychology’s Ethics Committee. Respondents in both samples provided consent via the first page of the electronic survey, where they had to indicate agreement before proceeding or were allowed to exit the survey. The consent form contained statements and directions regarding the nature of the study, the anonymity of the data, withdrawal both during and after participation, how the data would be stored in a coded form, how they could obtain the results of the study if required, and the intended use, length of storage and disposal of the data.

### Results

#### Exploratory Factor Analysis

The first step of the analysis was to determine the factor structure of the items. To allow any factor structure to emerge we used EFA in the first instance since, although we had a predicted EEA model, the underlying factor structure was unknown because of the inclusion of a number of items that may or may not have been relevant to the proposed EEA constructs. The number of participants (622) to variables (115) ratio exceeded the recommended minimum ratio needed for EFA of 5 to 1 (with a minimum number of participants of 150) [67, 68]. All items were subjected to maximum likelihood analysis (Kaiser-Meyer-Olkin measure of sampling adequacy = .94; Bartlett's test of sphericity, *x* ^2^ = 31329.51, *df* = 6555, *p* < .001).

The decision as to the number of factors to retain is imperative when carrying out EFA. Typically, this will be based on the K1 method (eigenvalues greater than one; [69]), a scree plot [70], and/or a parallel analysis of Monte Carlo simulations [71], the latter of which enables the researcher to compare the eigenvalues to those that might be expected from purely random data. The K1 approach is problematic and inefficient when it comes to determining the number of factors as it has a tendency to substantially overestimate the number of factors. Therefore, it is not recommended [72–74]. The K1 method applied to the current EFA, for example, suggests that 26 factors should be extracted as they are greater than one (21.90 to 1.02). Compared to the K1 approach, the scree test can be more accurate at determining the number of factors but it is also ambiguous, difficult to interpret, and subject to the researcher's expertise [73, 74]. With the current data, the scree test was difficult to interpret and ambiguous, with only a very close inspection suggesting a slight flattening of the plot at both the 11th and 15th eigenvalues, implying that 10- and 14-factor solutions could work at a conservative estimate. Various reports have suggested that parallel analysis is the most appropriate and accurate method for determining the number of factors, demonstrating the least variability and comparing favorably to the other methods [72–75]. Therefore, parallel analysis was used as the definitive guide in this study. The 11th eigenvalue (21.895. 6.728, 4.803, 3.509, 2.815, 2.399, 2.211, 1.950, 1.759, 1.684, 1.613) failed to exceed the 11th mean eigenvalue (1.995, 1.936, 1.893, 1.855, 1.821, 1.791, 1.761, 1.734, 1.708, 1.683 and 1.659) calculated from 1,000 generated datasets with 336 cases and 10 variables, suggesting that a 10-factor solution was appropriate. We explored other models (the 14-factor and 26-factor solutions) but the 10-factor solution was the one that could be considered theoretically consistent and the nearest to achieving a simple structure, i.e. a solution where items loaded most strongly onto one factor, and weakly onto the other factors [76].

Therefore, a 10-factor solution (Table 1) is reported using a promax rotation, as we expected the factors to be correlated, with delta set to 0. Meaningful loadings were assessed using the criteria of 0.32 ("poor"), 0.45 ("fair"), 0.55 ("good"), 0.63 ("very good"), and 0.71 ("excellent") [77, 78], and using these criteria 85 of the 115 items loaded above .32 on one of the factors, with 10 items loading above .32 on two or more factors. The item loadings are presented in Table 1. For the first four factors, we have presented all loadings above .63 (i.e. "very good" to "excellent") in bold, and have underlined those between .55 and .63. For the remaining six factors, we have simply underlined the highest-loading items.

**Table 1:** Maximum Likelihood Extraction with Promax Rotation of the Resilience Items.

Item	1	2	3	4	5	6	7	8	9	10
	1st factor									
1. Best effort no matter what (CD-RISC 10)	**.76**	-.03	-.01	-.09	-.06	-.03	.14	-.15	.16	.04
2. Determined (PRS 10)	**.70**	-.23	-.07	-.02	.22	-.07	.06	-.02	.03	-.08
3. Work to attain goals, no matter what roadblocks (CD-RISC 24)	**.70**	-.06	-.13	-.03	.09	.03	.10	-.23	.12	.06
4. You can achieve your goals, even with obstacles (CD-RISC 11)	**.63**	.11	-.09	-.01	-.09	.20	.03	-.15	.04	-.01
5. Self-discipline (PRS 14)	.61	-.09	.10	-.27	.26	-.06	.03	.20	-.03	.04
6. When things look hopeless, I don't give up (CD-RISC 12)	.58	.16	-.05	-.04	.03	.08	.01	-.10	-.03	-.01
7. Follow through with plans (PRS 1)	.55	-.01	-.01	-.12	.28	-.40	.13	.01	.22	-.01
8. Look forward to work (HS 17)	.54	.03	-.01	.11	-.17	-.06	.14	.14	-.17	.05
9. Under pressure, focuses and think clearly (CD-RISC 14)	.53	.20	.10	.09	-.01	-.08	-.07	-.01	-.01	.01
10. Interested in things (PRS 15)	.47	-.15	-.05	.10	.23	-.12	-.05	.26	.04	.13
11. Doing things that are worthwhile (HS 1)	.48	.01	.02	.02	-.09	.05	.17	.15	.15	.03
12. Prefer to take the lead in problem solving (CD-RISC 15)	.46	-.01	.04	.20	.03	-.04	.02	.05	-.12	-.24
13. Pride in your achievements (CD-RISC 25)	.39	.01	-.13	-.07	-.02	.28	.10	-.20	.31	.03
14. Eager to take up my life (HS30)	.37	.19	.08	.20	-.13	.05	.11	.05	.03	.17
15. Coping with stress strengthens (CD-RISC 7)	.36	.20	.09	-.01	.08	.23	-.04	-.18	-.05	-.02
16. Hand many things (PRS 9)	.34	.10	-.05	.01	.18	-.05	.01	.27	-.13	-.14
17. In control of your life (CD-RISC 22)	.33	.08	-.09	-.07	-.09	.20	-.10	.22	.10	-.14
	2nd factor									
18. Long time to get over set-backs (BRS 6)	.10	**-.95**	.12	.05	.05	.19	-.09	.04	-.01	.01
19. Hard to snap back (BRS 4)	.03	**-.90**	.12	.07	.09	.18	-.12	.08	.03	.04
20. Does not take a long time to recover (BRS 3)	-.11	**.90**	.07	.01	-.03	-.06	.11	-.04	-.01	.04
21. Come through difficult times (BRS 5)	-.07	**.82**	.13	-.06	-.02	-.07	.06	.09	-.03	.09
22. Bounce back quickly (BRS 1)	.08	**.71**	-.02	.05	.01	.03	.08	-.10	.02	.06
23. Hard time through stressful events (BRS 2)	-.03	**-.67**	.07	.11	.03	.10	.06	-.14	.19	.06
24. Can handle unpleasant feelings (CD-RISC 19)	.15	.47	.14	-.06	.16	.01	-.06	.08	-.11	-.13
25. Get over and recover (ER-89 2)	.07	.46	.02	.12	.07	-.12	.17	.06	-.10	-.01
26. Not easily discouraged (CD-RISC 6)	-.02	.35	-.04	.19	.17	-.06	-.05	-.04	.14	.13
27. Adapt to change	.24	.35	-.08	.11	.10	-.05	.04	-.18	.08	-.05
	3rd factor									
28. Working hard doesn't matter (HS 7)	-.15	.08	**.71**	.03	-.04	.04	.04	-.01	.12	-.15
29. Hard to imagine getting excited about working (HS 41)	-.13	.10	**.65**	.04	-.10	.09	.08	-.05	.21	-.07
30. Thinking of yourself as free leads to frustration (HS 24)	.09	-.09	**.65**	-.01	.12	-.09	.01	-.05	-.07	.04
31. It's hard to believe.. work helps society (HS 44)	.11	.03	**.64**	.06	-.20	-.01	-.09	.16	-.06	.10
32. Working people are manipulated (HS 9)	-.12	.08	.61	.12	.07	.09	.18	-.09	-.03	-.20
33. Belief in individuality (HS 37)	.12	.07	.57	.02	-.10	-.03	-.02	-.02	-.04	-.01
34. Impossible for me to change (HS 11)	-.12	-.05	.56	-.09	.06	.03	-.01	-.05	.25	-.02
35. Efforts accomplish nothing (HS 4)	-.09	-.12	.53	.03	.01	-.10	-.04	-.03	-.01	.06
36. Mistakes difficult to correct (HS 26)	.09	-.18	.53	.05	.01	.09	-.10	-.15	-.03	.09
37. Trying hard doesn't pay (HS 3)	-.01	-.08	.52	.01	-.05	-.11	.02	-.01	-.03	.01
38. Handle problems by not thinking about them (HS 28)	-.02	.08	.51	-.05	.03	.16	.08	.04	-.06	.23
39. Someone gets angry… no fault of mine (HS 43)	-.03	.14	.50	.03	-.02	.04	.13	.07	.04	.09
40. Ordinary work too boring (HS 45)	-.15	.13	.47	.15	.01	.06	.08	-.02	-.11	-.08
41. People never change their minds… have good judgment (HS 16)	.15	.03	.46	-.01	-.17	.24	.04	.14	-.02	.17
42. Can't prevent harm (HS 34)	.09	-.15	.40	.15	.01	.01	.02	-.03	-.04	.16
43. Politicians run lives (HS 18)	-.17	-.04	.38	.12	.05	.15	.23	-.13	.08	-.15
44. No use for theories not tied to facts (HS 38)	.06	.09	.34	-.03	.01	-.06	.13	-.02	-.06	-.10
45. Good athletes and leaders not made (HS 29)	-.07	.01	.34	-.05	.01	.23	-.02	.03	-.04	.19
46. Don't know own mind (HS 31)	-.13	-.14	.33	.14	.06	.03	.05	-.23	-.05	.27
47. Trying your best at work (HS 25)	.07	.05	-.32	.09	.07	.27	.31	.12	-.12	.23
	4th factor									
48. Changes in routine are interesting (HS 36)	-.16	.08	.10	**.73**	-.01	.03	-.09	-.08	.02	.12
49. Enjoy dealing with new and unusual situations (ER-89 3)	.08	-.02	.04	**.72**	.05	-.10	-.05	.01	-.07	-.01
50. Like new and different things (ER-89 11)	.07	-.10	-.04	**.68**	.08	-.03	.04	-.06	-.05	-.10
51. Like for uncertain or unpredictable (HS 33)	-.12	.02	.23	**.64**	.02	.03	-.17	.05	-.08	.14
52. More curious (ER-89 8)	.10	-.12	.11	.49	.17	-.04	.04	-.05	-.17	-.11
53. Different paths to familiar places (ER-89 7)	.13	.05	.21	.49	.04	-.06	.07	-.08	-.03	.01
54. Enjoy trying new foods (ER-89 5)	-.06	-.05	-.07	.49	.01	-.13	.04	-.03	.04	-.06
55. Variety in my work (HS 21)	-.03	-.12	-.17	.45	.12	.12	.26	-.11	.09	-.07
56. Energetic person (ER-89 6)	.17	-.09	.06	.38	-.13	.01	-.04	.20	.20	-.07
57. "Strong" personality (ER-89 13)	.18	-.04	.09	.34	.02	.05	.01	.20	.02	-.24
58. Favorable impression on people (ER-89 4)	.09	-.04	.05	.32	-.04	.02	.09	.14	.21	-.09
	5th factor									
59. Manage one way or another (PRS 2)	.10	.15	-.14	.01	.62	-.14	.08	-.04	.08	.01
60. Can be on own (PRS 5)	-.18	.01	-.19	-.08	.60	.10	.14	.04	-.19	-.07
61. Depend on myself (PRS 3)	-.02	.07	-.01	-.05	.59	.00	.21	.12	-.17	-.17
62. look at a situation in a number of ways (PRS 19)	.05	.10	.05	.15	.49	-.14	.01	.04	.12	.08
63. get through difficult times (PRS13)	.18	-.03	.05	-.04	.49	.01	-.03	.01	-.06	.03
64. somebody people generally rely on (PRS18)	.04	-.11	.01	.15	.47	-.12	.03	.13	.19	-.08
65. Keeping interested in things (PRS 4)	.12	-.18	-.11	.19	.45	-.02	.07	.14	.01	.05
66. make myself do things (PRS20)	.21	-.07	.01	.13	.42	-.18	.13	-.04	.03	.03
67. I can usually find my way (PRS23)	.19	.18	.02	.12	.39	-.06	-.04	.09	-.04	-.04
68. Take things in my stride (PRS7)	-.01	.30	.03	.01	.34	-.03	-.03	.25	.07	.06
69. Find something to laugh about (PRS 16)	-.10	.13	-.02	.18	.34	.03	-.06	.19	.27	.18
	6th factor									
70. Things happen for a reason (CD-RISC 9)	.04	-.17	.01	-.09	-.02	.58	.10	-.04	.15	.04
71. Meant to be (HS 10)	-.09	-.17	.25	-.04	.02	.54	.16	.07	.10	.07
72. Sometimes fate or God can help (CD-RISC 3)	.02	-.07	.13	-.08	-.17	.53	.01	.06	-.01	-.01
73. Success gives confidence (CD-RISC 5)	.29	.07	-.11	-.08	.08	.32	-.07	-.03	.12	-.04
74. You can always achieve your goals (HS 8)	.14	.07	-.29	.05	-.07	.32	.26	.08	-.07	.16
	7th factor									
75. Planning ahead can avoid problems (HS 2)	.27	.01	.02	.013	.03	.12	.44	-.13	.01	-.04
76. Answer questions before understanding (HS 20)	.05	.14	.09	-.03	.11	-.05	.35	-.07	.04	.01
77. New laws should never hurt wages (HS 12)	-.13	.09	.17	.08	.06	.15	.33	-.19	.17	-.08
78. "tried and true" ways (HS 6)	-.07	.07	.30	-.12	-.01	.25	.32	.05	.24	.01
	8th factor									
79. Friends with myself (PRS 8)	-.08	-.01	-.10	-.08	.24	.29	-.13	.44	.03	-.01
80. Life has meaning (PRS 21)	.06	-.02	-.14	-.01	.11	.28	-.01	.35	.26	-.02
81. Do not dwell on things (PRS 22)	-.04	.32	.16	-.05	.15	.07	-.11	.35	-.06	.03
	9th factor									
82. Close and secure relationship (CD-RISC 2)	.20	-.02	-.11	.04	-.05	.03	.02	-.01	.46	-.05
83. Want someone to care (HS 40)	.10	-.21	.11	-.04	-.05	.13	.11	-.05	.37	.08
84. Accomplished things in my life (PRS 6)	.27	-.07	-.07	-.10	.27	.09	.07	.09	.33	.09
85. Where to turn for help (CD—RISC 13)	.30	-.08	-.07	.01	-.08	.20	-.02	-.06	.32	-.05
	Items which load across factors, with highest loading on 1st factor									
86. I like challenges (CD-RISC 23)	.53	.08	-.01	.38	.01	-.12	-.07	-.14	-.07	-.01
87. Full of things that keep me interested (ER-89 12)	.43	-.11	.05	.36	-.09	-.18	-.05	.23	.15	.03
88. Can deal with whatever comes (CD-RISC 4)	.40	.33	.03	-.01	.12	.06	-.02	-.09	.04	-.07
89. You work to attain your goals (PRS24)	.38	.11	.03	-.05	.16	-.02	-.12	.34	.02	.07
90. Strong sense of purpose (CD-RISC 21)	.38	.02	-.02	-.04	-.15	.34	-.02	.10	.23	-.08
	Items which load across factors, with highest loading on 2nd factor									
91. Not easily discouraged by failure (CD-RISC 16)	.38	.43	.14	.01	-.08	.05	-.04	-.03	-.12	-.04
92. Tend to bounce back after illness or hardship (CD-RISC 8)	.32	.38	.07	-.02	.12	.09	-.07	-.14	.02	.08
	Items which load across factors, with highest loading on 3rd factor									
93. Hard to change a friend's mind (HS14)	.17	-.11	.45	-.06	-.09	.02	-.08	-.06	.08	.37
	Items which load across factors, with highest loading on 4th factor									
94. I don’t like to make changes (HS 5)	.12	-.02	.31	-.48	.16	-.05	.33	.10	-.03	.03
95. Bothers me when daily routine get interrupted (HS 27)	.24	-.03	.36	-.39	.08	.02	.37	-.05	-.03	.04
	Items failed to load above .32									
96. Respect rules (HS 32)	.21	.05	.08	-.18	-.07	.14	.30	-.02	.25	.22
97. People I meet are likeable (ER-89 9)	.08	-.02	-.08	.30	-.03	-.04	.05	.01	.21	.14
98. Generous with my friends (ER-89 1)	-.02	.03	-.14	.17	.11	-.06	.15	-.04	.18	-.05
99. Life is interesting and exciting (HS 39)	.27	.16	.09	.29	-.19	.01	.05	.24	.14	.11
100. One day at a time (PRS 12)	-.24	.15	.17	-.05	.27	.14	-.04	.14	.12	.20
101. Think carefully before acting (ER-89 10)	.24	-.03	.12	-.12	.09	-.05	.27	.06	.07	-.13
102. Exciting to learn about myself (HS 15)	.02	-.08	-.09	.21	.13	.30	.23	-.08	.07	-.01
103. People should get full support from society (HS 35)	.09	-.06	-.09	.11	.07	.16	.25	-.12	.06	.04
104. I know when to seek help (HS 19)	.27	-.02	-.10	.05	.01	.16	.16	.03	.05	.05
105. Get over my anger quickly (ER-89 14)	-.17	.29	-.04	.24	.09	-.03	.05	.11	.03	.21
106. When I make plans, I can make them work (HS 13)	.29	.22	.07	.12	.01	.01	.28	.08	.01	.02
107. Have to act on a hunch (CD-RISC 20)	.15	.01	.13	.10	.03	.18	-.03	-.15	-.01	.03
108. People listen carefully to what I say (HS 22)	.16	-.01	.05	.28	-.03	.06	.18	.20	-.02	-.15
109. Seldom wonder what the point of it is (PRS11)	-.04	-.01	-.02	-.03	.07	-.01	-.05	.24	-.03	.01
110. What happens tomorrow depends on today (HS 42)	.15	-.06	.12	.09	.16	.15	.24	-.13	-.05	.04
111. Belief gets me through hard times (PRS17)	.16	.01	.04	-.04	.29	.31	-.16	.31	-.07	.01
112. Think of self as strong person (CD-RISC 17)	.29	.25	-.01	.08	.09	.21	-.11	.05	-.10	-.15
113. Okay if people don’t like me (PRS 25)	-.03	.22	.01	-.05	.27	.05	-.01	.25	-.17	-.16
114. Daydreams are more exciting (HS 23)	-.19	-.16	.26	.09	.18	.19	.05	-.22	-.17	.05
115. Make unpopular or difficult decisions (CD-RISC 18)	.24	.02	.10	.11	.04	.08	-.04	-.04	-.22	-.18

Key: PRS = Psychological Resilience Scale; CD-RISC = Connor-Davidson Resilience Scale; BRS = Brief Resilience Scale; HS = Hardiness Scale; ER-89 = Ego Resilience Scale.

NB: For copyright reasons the items have been truncated. The items belonging to the Connor-Davidson scale (CD-RISC) are truncated as in Connor and Davidson (2003). Full items are available via the original articles and for the CD-RISC via www.connordavidson-resiliencescale.com/.

When considering these loadings, the first four factors emerge as having items with "very good" to "excellent" loadings (i.e. above .63), with the first, second, and fourth factors reflecting the proposed EEA model of resilience. The first factor (19.04% of the variance) contains four items loading above .63; three of the items are from the CD-RISC scale and one is from the Psychological Resilience Scale. These items reflect someone who reports giving their best effort, being determined, and working and trying their hardest to attain their goals, despite obstacles. Therefore, this factor would be the proposed ecological resilience factor, with the emphasis here on seeking to maintain one's key function and identity (e.g. "determined", "best effort") while simultaneously monitoring one's own structure and processes that govern one's system of behavior used to accommodate or resist any disturbance (e.g. "working to attain goals, no matter what the roadblocks"). The items that load above .63 on Factor 2 (5.85% of the variance) are all the items from the Brief Resilience Scale, and they reflect the extent to which an individual is relatively easily able to bounce back from stressful and difficult times. As such, this factor reflects the proposed engineering resilience factor, a system featuring ability to and speed of recovery (e.g. "does not take a long time to recover"). The fourth factor (3.05% of the variance) has four items loading above .63; two of the items are from the Hardiness Scale and two from the ER89 scale. These items reflect someone who reports finding enjoyment and interest in change and differences, uncertainty, and unusual situations. Therefore, this factor reflects the proposed adaptive resilience factor, which captures systems that persist and resist disturbance through a willingness to adapt continually (e.g. "changes in routine are interesting") and accept variety in key functions and processes (e.g. "I enjoy dealing with new and unusual situations").

It is worth noting the other factors that emerged from the analysis. In terms of factors that have items that load above .63, Factor 3 (4.18% of the variance) is defined by items from the commitment subscale of the Hardiness Scale and contains attitudinal statements rather than core descriptions of the individuals' own behaviors (e.g. "I find it hard to believe people who say their work helps society", or "most working people are simply manipulated by their bosses''). More strikingly, most of these items are specific to attitudes around certain work situations. Therefore, the confluence of these attitudinal statements in this analysis falls outside the current aims of the study, namely to assess trait resilience, and therefore it is argued that this factor should be ignored. The remaining factors (5 to 10, explaining from 2.45% to 1.47% of the variance) do not contain items that load above .63, and therefore their interpretation and replication may be problematic. However, in terms of what these remaining factors may reflect, the items that load most highly on the fifth factor describe self-reliance, while those that load most highly on the sixth factor reflect an acceptance of fate. In terms of Factors 7 to 10, the number of items that load on them reaches a minimum of three items on the factor for interpretation, but they do not load highly enough to allow for the successful identification of these factors [79, 80]. Though 20 of the items do not load on any factor at all, inspection of the content of these items suggests, in terms of face validity, that the majority do not necessarily assess trait resilience but other trait behaviours, for example sociability ("I am generous with my friends"; "life is interesting and exciting"; "people I meet are likeable"), leadership ("people listen carefully to what I say"; "I make unpopular or difficult decisions"), self-reflection ("it is exciting to learn about myself"), cautiousness ("I think carefully before acting"), or anger regulation ("I get over my anger quickly"). Therefore, it is argued that this failure of a number of items to load on any factor is not a concern in the current study.

The correlations between the three proposed EEA factors were found to be .55 (engineering and ecological resilience factors), .49 (engineering and adaptive resilience factors) and .50 (ecological and adaptive resilience factors), suggesting that the overall factors from this analysis share no more than 30% common variance.

In light of these findings we make two proposals: first, that three factors from the EFA (the first, second and fourth factors) can be used to measure the EEA dimensions of trait resilience; second, that the best four items from each of these factors can be used to measure each facet, as their loadings on these factors represent a "very good" or better assessment.

The mean (noting the different response formats for items from different measures), standard deviation, skewness, and kurtosis statistics were calculated for each of the suggested 12 EEA trait resilience items (Table 2). The latter two statistics are used to consider the asymmetry of the distribution of responses to these items. Both the skewness and kurtosis statistics for the 12 items fall within criteria specified across a range of statistical analyses, namely +/-1 representing "very good" symmetry of a normal univariate distribution, values of +/-2 representing "acceptable" symmetry, and skewness > 2 and kurtosis > 7 representing a concern around symmetry for a normal univariate distribution [81–83]. Finally, the Cronbach's alpha [84] coefficients for the proposed four-item scales (adaptive, α = .77, ecological, α = .73, and engineering, α = .83) exceed the internal reliability criterion of α > .70 as "good" [85, 86].

**Table 2:** Mean, Standard Deviation, Skewness and Kurtosis Statistics for the EEA Trait Resilience Items (EFA).

Item	Mean	SD	Skewness	Kurtosis
1. Long time to get over set-backs (BRS 6)	2.86	1.0	.11	-.84
2. Hard to snap back (BRS 4)	2.86	1.0	.10	-.97
3. Does not take a long time to recover (BRS 3)	3.22	1.0	-.14	-.93
4. Come through difficult times (BRS 5)	3.10	1.0	-.11	-.89
5. Best effort no matter what (CD-RISC 10)	3.73	.9	-.37	-.07
6. Determined (PRS 10)	5.56	1.2	-.65	.10
7. Work to attain goals, no matter what roadblocks (CD-RISC 24)	3.82	.8	-.44	.04
8. You can achieve your goals, even with obstacles (CD-RISC 11)	3.85	.8	-.45	-.05
9. Changes in routine are interesting (HS 36)	1.40	.8	.14	-.46
10. Dealing with new and unusual situations (ER-89 3)	2.74	.9	-.24	-.57
11. New and different things (ER-89 11)	3.02	.8	-.27	-.66
12. Like for uncertain or unpredictable (HS 33)	1.08	.9	.40	-.65

Key: PRS = Psychological Resilience Scale; CD-RISC = Connor-Davidson Resilience Scale; BRS = Brief Resilience Scale; HS = Hardiness Scale; ER-89 = Ego Resilience Scale.

NB: In terms of noting the means, there are different possible ranges of scores for items from different scales. For copyright reasons the items have been truncated. The items belonging to the Connor-Davidson scale (CD-RISC) are truncated as in Connor and Davidson (2003). Full items are available via the original articles and for the CD-RISC via www.connordavidson-resiliencescale.com/.

### Confirmatory Factor Analysis

The mean, standard deviation, skewness, kurtosis, and item inter-correlation statistics were calculated for each of the suggested 12 EEA trait resilience items (Table 3) using the new four-point response format for all items so as to standardize the rating scales. Skewness and kurtosis values for 10 of the 12 items fell within the aforementioned skewness and kurtosis values of +/-1, representing "very good" symmetry of a normal univariate distribution. For two items, either the skewness or the kurtosis value exceeded +/-1: "Determined" (PRS 10) and "Have come through difficult times". However, for both these items the skewness and kurtosis values fell within the criteria of +/-2, representing "acceptable" symmetry of a normal univariate distribution, not contravening the aforementioned criteria for concern [81].

**Table 3:** Mean, Standard Deviation, Skewness, Kurtosis and Item Intercorrelation Statistics for the EEA Trait Resilience Items (CFA).

Item	Mean	SD	Skew	Kurtosis	1	2	3	4	5	6	7	8	9	10	11	12
1. Long time to get over set-backs (BRS 6) (R)	2.33	.93	-.04	-.99	-	.72**	.57**	.58**	.10	.25**	.32**	.26**	.18*	.14	.22**	.21**
2. Hard to snap back (BRS 4) (R)	2.19	.93	.16	-.99		-	.66**	.59**	.11	.22**	.30**	.25**	.12	.15	.17*	.11
3. Does not take a long time to recover (BRS 3)	2.27	.87	-.13	-.99			-	.514**	.20*	.23**	.32**	.30**	.07	.18*	.13	.07
4. Come through difficult times (BRS 5)	2.22	.89	.01	-1.01				-	.08	.12	.25**	.13	.18*	.19*	.06	.24**
5. Best effort no matter what (CD-RISC 10)	2.93	.81	-.30	-.52					-	.63**	.61**	.65**	.02	.23**	.09	.04
6. Determined (PRS 10)	3.27	.91	-1.25	.84						-	.67**	.67**	-.03	.16*	.16*	-.02
7. You work to attain your goals (CD-RISC 24)	2.99	.98	-.78	-.32							-	.66**	-.03	.18*	.17*	.05
8. You can achieve your goals (CD-RISC 11)	2.91	.87	-.42	-.52								-	.02	.26**	.25**	.15
9. Changes in routine are interesting (HS 36)	2.62	.79	.05	-.48									-	.59**	.55**	.67**
10. Dealing with new and unusual situations (ER-89 3)	2.77	.87	-.47	-.35										-	.67**	.59**
11. New and different things (ER-89 11)	2.90	.82	-.46	-.21											-	.49**
12. Like for uncertain or unpredictable (HS 33)	2.43	.85	-.01	-.59												-

** p < .01;

* p < .05;

(R) = Reversed Item

Key: PRS = Psychological Resilience Scale; CD-RISC = Connor-Davidson Resilience Scale; BRS = Brief Resilience Scale; HS = Hardiness Scale; ER-89 = Ego Resilience Scale.

**Fig 1:**
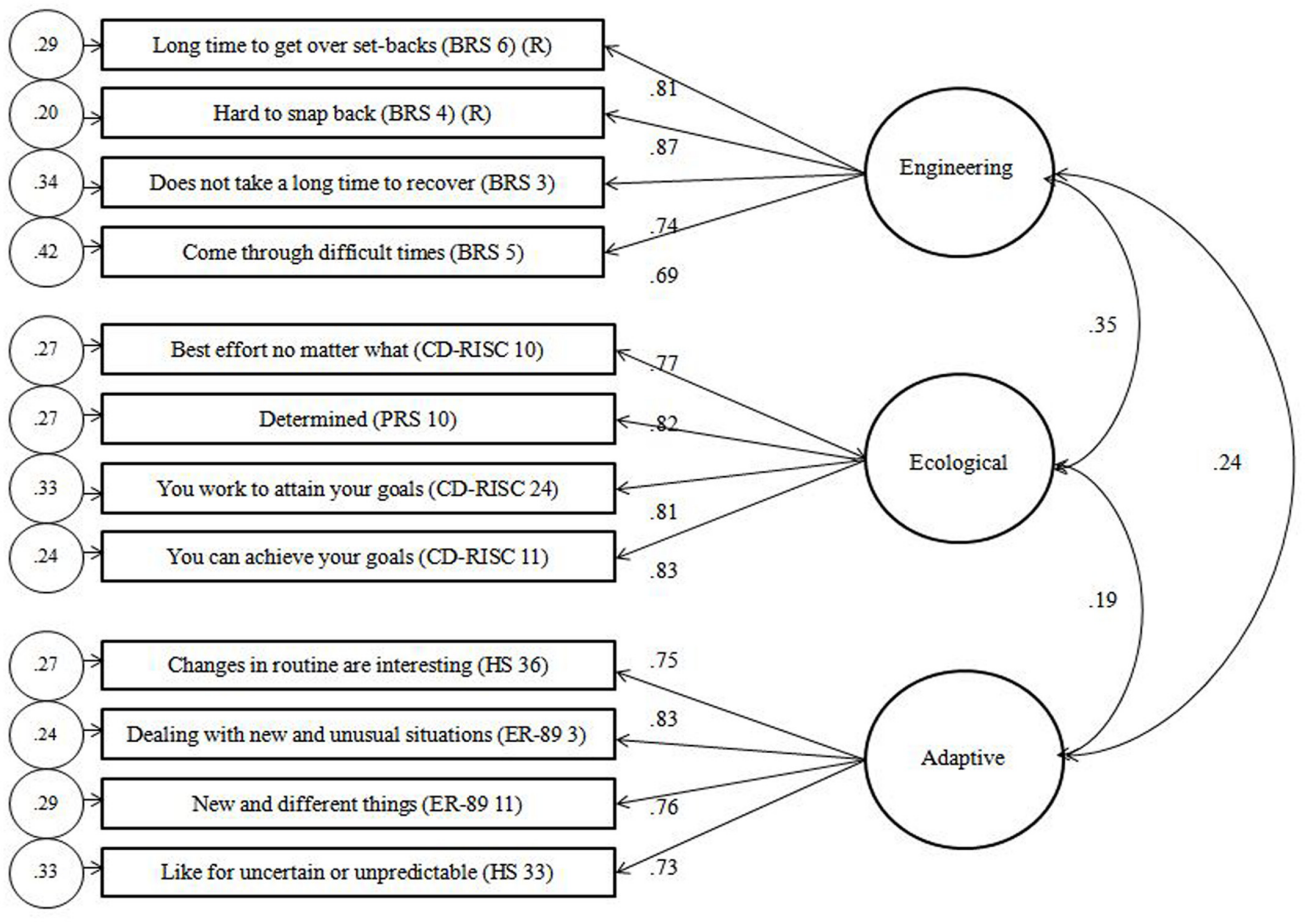
Confirmatory Factor Analysis of the EEA Trait Resilience Items.

CFA was performed with AMOS 20 software on the data collected from Sample 2 using the 12-item scale based on the three-factor solution suggested by the EFA (Fig 1). To assess goodness of fit, we report the relative chi-square (CMIN/DF) alongside the chi-square and degrees of freedom, the goodness of fit index (GFI), comparative fit index (CFI), non-normed fit index (NNFI), root mean square error of approximation (RMSEA), and standardized root mean square residual (SRMR), on the recommendations of Hoyle [87], Hu and Bentler [88], and Kline [89]. Acceptable fit is indicated by CMIN/DF of less than 2 or 3, GFI, CFI, and NNFI of at least .90, RMSEA index from .05 to .08, and SRMR less than .08 [78, 87–90]. As it is useful to demonstrate the incremental value of proposed models [91], we compared the three-factor interpretation of the data against a one-factor model, proposing that all 12 items could load on one factor reflecting an underlying latent factor of trait resilience.

For both the one- and three-factor models, fit statistics for the parent/congeneric, tau equivalence, and parallel models are presented (Table 4). The fit statistics for the three-factor model meet the aforementioned criteria of an acceptable fit to the data while the fit statistics for the one-factor model do not. For the three-factor model, the parent/congeneric model presents a significantly better fit of the data than either the tau equivalence model (Δ CMIN = 25.19, Δ *df* = 11, *p* = .009) or the parallel model (Δ CMIN = 39.39, Δ *df* = 22, *p* = .013). However, the tau equivalence and parallel models fit the data equally well (Δ CMIN = 14.20, Δ *df* = 11, *p* = .22). For the one-factor model, the parent/congeneric model presents a significantly better fit of the data than either the tau equivalence model (Δ CMIN = 67.05, Δ *df* = 11, *p* < .001) or the parallel model (Δ CMIN = 73.34, Δ *df* = 22, *p* < .001). However, again, the tau equivalence and parallel models fit the data equally well (Δ CMIN = 6.29, Δ *df* = 11, *p* = .85). Across the three models, the three-factor model represents a significantly better fit of the data than the one-factor model (parent/congeneric, Δ CMIN = 520.99, Δ *df* = 3, *p* < .001; tau equivalence, Δ CMIN = 564.97, Δ *df* = 3, *p* < .001; parallel, Δ CMIN = 554.94, Δ *df* = 3, *p* < .001). Together, these findings suggest that the fit statistics for the parent/congeneric three-factor model demonstrate an adequate fit against goodness of fit criteria, compare favourably to the fit statistics obtained for a proposed one-factor model, and present a significantly better fit of the data within a nested model.

**Table 4:** Confirmatory Factor Analysis Fit Statistics for the 1 Factor and 3 Factor Models Proposed for the EEA trait resilience items.

	x2	df	CMIN/DF	GFI	CFI	NNFI	RMSEA	SRMR
	One factor							
Parent/congeneric measurement	619.55	54	11.47	.56	.43	.30	.25	.21
Tau equivalence measurement	686.60	65	10.56	.51	.37	.36	.24	.20
Parallel measurement	692.89	76	9.12	.50	.38	.46	.22	.20
	Three factor							
Parent/congeneric measurement	98.56	51	1.93	.91	.95	.94	.075	.06
Tau equivalence measurement	121.63	62	2.00	.89	.94	.93	.077	.07
Parallel measurement	137.95	73	1.89	.87	.93	.94	.073	.07

A full range of scores (from 4 to 16) was found for each of the scales, with the mean and standard deviation scores as follows for each subscale: engineering resilience (*M* = 9.02, *SD* = 3.0), ecological resilience (*M* = 12.11, *SD* = 3.1), and adaptive resilience (*M* = 10.73, *SD* = 2.8). Furthermore, the skewness and kurtosis values for the three scales fall within the criteria of +/-1, representing "very good" symmetry of a normal univariate distribution: engineering resilience (skewness = .19; kurtosis = -.40), ecological resilience (skewness = -.84; kurtosis = .20) and adaptive resilience (skewness = -.47; kurtosis = .24). Additionally, the Cronbach's alpha coefficients for the scales (engineering resilience, α = .86, ecological resilience, α = .88, and adaptive resilience, α = .85) exceed the aforementioned internal reliability criterion of α > .70 = "good". Finally, no sex differences (*t* = < 1.29, *df* = 166, *p* > .12) or associations with age (*r* = < .06, *df* = 168, *p* > .46) were found for any of the EEA items or scales.

## Study 2

The aim of Study 2 was to consider EEA trait resilience within wider trait psychology, and its relationship to well-being, by collating the facets of EEA trait resilience with measures of personality, coping, and well-being.

### Materials and Method

#### Sample

The sample comprised 256 respondents (74 men, 182 women) aged from 18 to 36 years (*M* = 19.78 years, *SD* = 2.7). The participants were volunteers from the university experiment participation scheme described in Study 1.

#### Materials

The respondents were given the 12-item measure of EEA resilience developed in Study 1 with the aforementioned standardized four-point scale response format (1 = *Strongly disagree* to 4 = *Strongly agree*). In addition, the respondents were given six further scales, to measure personality, coping, and well-being.

Personality was assessed via the 60-item *Short Five* [92]. This scale measures 30 facets of the five-factor model of personality, the factors being neuroticism, extraversion, openness to experience, conscientiousness, and agreeableness. Responses are scored using a seven-point scale, ranging from -3 (*Completely disagree*) to +3 (*Completely agree*). The Short Five has demonstrated good reliability statistics, correlations above .8 with longer counterpart measures of the five-factor model of personality across Estonian, Finnish, English, and German samples, and a factor structure similar to that of the Revised *NEO* Personality Inventory [92].

Coping was measured via the *Functional Dimensions of Coping Scale* [93], which assesses approach, avoidance, reappraisal, and emotional regulation. To adapt the focus of the measurement of coping styles from one particular stressful event to typical coping traits used across many situations, we changed the instructions to those used with the COPE scale [94], which asks the individual to consider "what activities you usually do when you are under a lot of stress". Then, in accordance with the Functional Dimensions of Coping Scale instructions, the respondents were asked to answer each item by indicating to what extent these activities helped them (e.g. "allow you to directly deal with the problem"), using a seven-point scale, ranging from 0 (*Not at all*) to 6 (*Very much so*). The coefficient alphas have been shown to exceed α = .70 for the original version of the subscales [93]. The validity of the Functional Dimensions of Coping Scale has been shown by the fact that it is uncorrelated with social desirability and correlated in theoretically meaningful ways with self-reported coping behaviors and individual differences in health anxiety [93].

To measure the key indices of well-being in terms of mental health we used three measures designed to assess three domains of well-being: hedonic or subjective well-being (assessment of current pleasure versus pain experiences; [95]), eudemonic or psychological well-being (reflecting the longer-term engagement and meaning derived from life challenges; [95]), and current experiences of health issues. To measure subjective well-being, we measured three facets to reflect Diener's definition of subjective well-being that deals with lower negative affect, greater satisfaction with life, and positive affect [96–98]. We did this by using the *Positive and Negative Affect Scales* (PANAS; [99]) and the *Satisfaction with Life Scale* (SWLS; [100]). The PANAS is a 20-item scale that comprises two subscales assessing positive and negative mood states that are rated on a 1 (*Very slightly or not at all*) to 5 (*Extremely*) scale for the past week. The SWLS is a five-item scale using a seven-point response format from 1 (*Strongly disagree*) to 7 (*Strongly agree*). To measure eudemonic well-being, we used the *Scales of Psychological Well-being* [101]. This measure consists of 18 items that encompass six dimensions of psychological well-being (three items per dimension: autonomy, environmental mastery, positive relations with others, personal growth, purpose in life, and self acceptance). We asked the participants to respond using a six-point Likert scale ranging from 1 (*Strongly disagree*) to 6 (*Strongly agree*).

To measure well-being in terms of physical health we used the *Physical Health Questionnaire* (PHQ) [102]. This 14-item scale comprises four subscales assessing somatic symptoms: gastrointestinal problems (four items; e.g. "How often have you suffered from an upset stomach (indigestion)?"), headaches (three items; e.g. "How often have you experienced headaches?"), sleep disturbances (four items; e.g. "How often have you had difficulty getting to sleep at night?"), and respiratory illness (three items; e.g. "When you have a bad cold or flu, how often does it last longer than it should?"). Responses are scored on a seven-point scale from 1 (*Not at all*) to 7 (*All of the time*). Cronbach’s alpha values for the subscales have been shown to be satisfactory, ranging from α = .70 (for the respiratory illness subscale) to α = .90 (for the gastrointestinal problems subscale) [102]. For the purposes of this study, we computed an overall physical health score.

In addition, to examine the contribution of EEA trait resilience to well-being over time, a follow-up study was administered in which, of the original 256 respondents, 101 (25 males, 74 females) completed the PANAS, SWLS, Scales of Psychological Well-being and PHQ, four months after the original administration of the survey.

In a separate study, to test the stability of EEA trait resilience as a trait, 89 of the original 256 respondents (22 males, 67 females) completed the EEA trait resilience measure five months after the original administration.

#### Ethical Consent

The study procedures received ethical approval from the University of Leicester's School of Psychology's Ethics Board as outlined in Study 1.

### Results

To assess subjective well-being, we aimed to calculate a subjective well-being factor score from the three subscales contained within the PANAS and SWLS. Parallel analysis suggested a one-factor structure with the second eigenvalue emerging from a maximum likelihood extraction (1.833, .728) failing to exceed the second mean eigenvalue (1.097, 1.000) calculated from 1,000 generated datasets with 256 cases and three variables. The three scales loaded on the unrotated factor as follows: SWLS, .93; positive affect, .52; negative affect, -.53. This suggested that an overall factor score could be computed from these three variables.

Reliability statistics and mean scores were computed for all the remaining measures (see Table 5). The alpha coefficients for the scales in our sample exceeded the aforementioned acceptable internal reliability criteria of α > .7 = "good". For the 89 respondents who completed the EEA trait resilience measure twice, the interclass correlation coefficients (engineering, ICC = .71; ecological, ICC = .80; adaptive, ICC = .77) were all above the .6 minimum threshold suggested by Chinn [103], with two of the scales above the threshold of .75, representing "excellent" reliability [104].

**Table 5:** Cronbach's alpha, Means, SDs, and Zero-Order Correlations between EEA Trait Resilience, Personality, Coping and Well-being.

Scale	α	Mean (SD)	2	3	4	5	6	7	8	9	10	11	12	13	14	15
1. Engineering resilience	.81	8.58 (3.1)	.34**	.24*	-.61**	.21**	.06	.10	.13*	.11	.09	.28**	.16*	.35**	.36**	-.41**
2. Ecological resilience	.80	12.31 (2.4)	-	.29**	-.43**	.44**	.21**	.15*	.50**	.27**	-.04	.27**	.32**	.59**	.64**	-.42**
3. Adaptive resilience	.77	9.89 (2.6)		-	-.25**	.41**	.37**	.06	.05	.22**	.08	.27**	.28**	.26**	.26**	-.10
4. Neuroticism	.89	-4.46 (13.1)			-	-.46**	-.11	-.14*	-.33**	-.15*	.01	-.35**	-.21**	-.58**	-.62**	.56**
5. Extraversion	.84	8.06 (11.0)				-	.38**	.15*	.22**	.12*	.01	.23**	.27**	.53**	.57**	-.33**
6. Openness	.81	12.81 (10.4)					-	.40**	.27**	.11	-.10	.18**	.21**	.23**	.42**	-.14*
7. Agreeableness	.75	12.78 (8.9)						-	.38**	.06	-.11	.12	.13*	.20**	.33**	-.19**
8. Conscientiousness	.86	12.60 (10.6)							-	.27**	-.16**	.20**	.29**	.36**	.57**	-.28**
9. Approach coping	.85	17.21 (5.1)								-	-.10	.54**	.80**	.22**	.23**	-.10
10. Avoidance coping	.75	15.02 (5.0)									-	.23**	-.08	-.02	-.14*	.03
11. Emot Reg coping	.81	13.62 (3.6)										-	.60**	.34**	.30**	-.19**
12. Reappraisal coping	.87	23.03 (6.4)											-	.27**	.33**	-.14*
13. SWB	N/A	N./A												-	.69**	-.53**
14. PWB	.85	79.93 (11.5)													-	-.54**
15. Physical health	.93	41.76 (15.4)														-

* *p* < .05;

** *p* < .01

Key: Emot Reg = Emotional Regulation; SWB = Subjective Well-being; PWB = Psychological Well-being.

Table 5 also shows the zero-order correlations between the measures. To assess the importance of these correlations, we used a conventional frame of reference, with *r* > = .5 representing a large effect size, .3 ≤ *r* < .5 representing a moderate effect size, and .1 ≤ *r* < .3 representing a small effect size [105, 106], with the criterion of a moderate effect size deemed to be the minimum at which the findings can be considered to be of practical significance [106, 107]. In terms of engineering resilience, significant and large effect size associations occurred for low neuroticism, and significant and moderate effect size associations occurred for all the well-being variables, namely, subjective well-being, psychological well-being, and physical health. In terms of ecological resilience, significant and large effect size associations occurred for higher psychological well-being, subjective well-being, and conscientiousness; significant and moderate effect size associations occurred for lower neuroticism, and for higher extraversion, reappraisal coping, and physical health. Finally, in terms of adaptive resilience, significant and moderate effect size associations occurred for higher extraversion and openness to experience.

To further examine the relationship between the EEA trait resilience, and personality and coping, we ran a series of standard multiple regressions to examine which aspects of personality and coping predict unique variance in each of the EEA trait resilience facets. We controlled for sex and age in these relationships given previous reports of gender and age-based differences in personality, coping, and well-being [108, 109]. The variance inflation factors (VIFs) and tolerance factors for the predictor variables were no larger than 3.48 and no smaller than .29 respectively. Therefore, they did not contravene the threshold values for VIF of at least 5 and tolerance statistics of less than .2 that are used to suggest collinearity between independent variables [110].

The results of the regression analysis for each trait resilience variable are presented in Table 6. For each of the regressions, sex, age, personality, and coping demonstrate statistical significance in predicting trait resilience (engineering, *F* [11, 244] = 15.06, *r* = .64; *r* ^2^ = .4, adj *r* ^2^ = .38, *p* < .001; ecological, *F* [11, 244] = 15.33, *r* = .64; *r* ^2^ = .41; adaptive, *F* [11, 244] = 9.56, *r* = .55; *r* ^2^ = .3, adj *r* ^2^ = .27, *p* < .001). Lower neuroticism accounts for unique variance in engineering resilience. Lower neuroticism, and higher extraversion and conscientiousness, account for unique variance in ecological resilience. Lower neuroticism, and higher extraversion, openness to experience, and conscientiousness, predict unique variance in adaptive resilience. However, adopting the criteria to identify associations of a moderate (or greater) effect size (e.g. β > .3) in predicting facets of the EEA trait resilience model, we can see that the coefficients of a moderate effect size indicate lower neuroticism for engineering resilience, conscientiousness for ecological resilience, and openness to experience for adaptive resilience.

**Table 6:** Regression Analysis with Facets of EEA Trait Resilience Used as Dependent Variables, and Sex, Age, Personality, and Coping Used as Predictor Variables.

	Engineering resilience				Ecological resilience				Adaptive resilience			
	B	β	t	Sig	B	β	t	Sig	B	β	T	Sig
1. Sex	-.61	-.09	-1.78	.076	-.19	-.04	-.73	.468	-.23	-.04	-.73	.465
2. Age	.07	.06	1.15	.250	.01	.01	.22	.827	.07	.07	1.32	.189
3. Neuroticism	-.15	-.65	-10.62	.001	-.03	-.17	-2.76	.006	-.03	-.13	-2.04	.042
4. Extraversion	-.03	-.09	-1.50	.136	.06	.27	4.43	.000	.06	.24	3.63	.001
5. Openness	.01	.01	.10	.920	-.01	-.01	-.15	.878	.08	.30	4.73	.001
6. Agreeableness	.02	.07	1.15	.251	-.02	-.06	-1.00	.321	-.02	-.07	-1.15	.253
7. Conscientiousness	-.03	-.11	-1.87	.062	.09	.38	6.52	.001	-.04	-.14	-2.27	.024
8. Approach coping	-.02	-.04	-.42	.673	.04	.08	.91	.364	.05	.09	1.01	.316
9. Avoidance coping	.05	.09	1.58	.116	.01	.03	.51	.614	.06	.11	1.88	.061
10. Emot Reg coping	.03	.04	.52	.601	.01	.01	.09	.928	-.01	-.01	-.08	.939
11. Reappraisal coping	.04	.08	.85	.394	.01	.04	.41	.681	.04	.10	1.04	.300

Key: Emot Reg = Emotional Regulation.

Further, to examine whether the EEA trait resilience demonstrates incremental value over existing models of personality and coping in predicting well-being, we ran a series of two-step multiple regressions to examine which aspects of resilience (predictor variables in step 2) predict well-being (dependent variables) after controlling for sex, age, personality, and coping (predictor variables in step 1). Again,VIFs and tolerance factors for the predictor variables are no larger than 3.59 and no smaller than .29 respectively. Therefore, they do not contravene the aforementioned threshold values for VIFs of at least 5 and for tolerance statistics of less than .2 which are used to suggest multicollinearity.

The results of the regression analysis for each well-being variable are presented in Table 7. In step 1, sex, age, personality, and coping demonstrate statistical significance in predicting each type of well-being (subjective well-being, *F* [11,244] = 20.67, *r* = .70, *r* ^2^ = .48, adj *r* ^2^ = .46, *p* < .001; psychological well-being, *F* [11,244] = 43.38, *r* = .81, *r* ^2^ = .66, adj *r* ^2^ = .65, *p* < .001; physical health, *F* [11,244] = 11.69, *r* = .59, *r* ^2^ = .35, adj *r* ^2^ = .32, *p* < .001). Lower age and neuroticism, and higher extraversion, conscientiousness, and emotional regulation account for unique variance in subjective well-being. Lower neuroticism and higher extraversion, openness to experience, and conscientiousness account for unique variance in psychological well-being. Higher neuroticism accounts for unique variance in worse physical health. In step 2, the inclusion of the EEA trait resilience scales cause a statistically significant change in *R* ^2^ for all aspects of well-being (subjective well-being, Δ*R* = .07, *p* < .001; psychological well-being, Δ*R* = .04, *p* < .001; physical health, Δ*R* = .04, *p* = .001). Ecological resilience accounts for unique variance in subjective well-being, psychological well-being, and physical health. Adaptive resilience accounts for unique variance in physical health.

**Table 7:** Regression Analysis with Subjective Well-being, Psychological Well-being and Physical Health as Dependent Variables, and Sex, Age, Personality, Coping and EEA Trait Resilience Used as Predictor Variables.

	Subjective Well-being				Psychological Well-being				Physical Health			
	B	β	T	Sig	B	β	t	Sig	B	β	t	Sig
Step 1												
1. Sex	.09	.04	.909	.364	.04	.01	.26	.797	-.06	-.01	-.19	.847
2. Age	-.05	-.13	-2.73	.007	-1.40	-.06	-1.46	.145	3.33	.10	1.87	.063
3. Neuroticism	-.02	-.35	-6.09	.001	-.32	-.37	-8.01	.001	.58	.49	7.76	.001
4. Extraversion	.03	.31	5.47	.001	.27	.25	5.49	.001	-.11	-.08	-1.26	.208
5. Openness	.01	.01	.15	.878	.17	.16	3.47	.001	.01	.01	.04	.971
6. Agreeableness	.01	.04	.80	.428	.07	.06	1.30	.196	-.17	-.10	-1.63	.105
7. Conscientiousness	.01	.14	2.56	.011	.33	.31	6.93	.001	-.10	-.07	-1.12	.263
8. Approach coping	.02	.08	1.07	.279	-.03	-.01	-.21	.833	-.09	-.03	-.32	.749
9. Avoidance coping	-.01	-.04	-.86	.391	-.15	-.06	-1.55	.123	-.01	-.01	-.08	.938
Emotional regulation coping	.03	.13	2.00	.047	-.02	-.01	-.11	.912	.14	.03	.43	.666
Reappraisal coping	-.01	-.07	-.79	.432	.12	.07	.94	.349	.06	.03	.27	.788
Step 2												
1. Engineering resilience	-.01	-.02	-.38	.705	.01	.01	.05	.962	-.53	-.11	-1.61	.110
2. Ecological resilience	.13	.35	5.99	.001	1.25	.26	5.63	.001	-1.28	-.20	-3.00	.003
3. Adaptive resilience	-.01	-.02	-.26	.793	-.31	-.07	-1.67	.095	.79	.14	2.23	.027

Finally, we considered whether any of the trait resilience facets predicted aspects of well-being (subjective, psychological, and physical health well-being) at a second time point, five months after the original administration of the survey, controlling for the respective well-being at the original time (Time 1), among a subsample of 101 participants who agreed to participate in a follow-up study. No significant differences were found between Times 1 and 2 for the subjective well-being factor score (*t* = -.81, *p* = .422), psychological well-being (*t* = -.81, *p* = .420), or physical health (*t* = -.97, *p* = .335) for the overall sample. To assess subjective well-being at Time 2 (five months on), we again calculated a factor score from the three scales derived from PANAS and SWLS. Parallel analysis suggested a one-factor structure, with the second eigenvalue (1.930, .726) failing to exceed the second mean eigenvalue (1.099, 1.000) calculated from 1,000 generated datasets with 256 cases and three variables. The three scales load on this factor as follows: SWLS, .98; positive affect, .63; negative affect, -.46.

We then ran three two-step multiple regressions, with each well-being measure at Time 2 used as a dependent variable, the respective measure of well-being at Time 1 used as the predictor variable in step 1, and the EEA trait resilience scales at Time 1 used as the predictor variable in step 2. For the subjective and psychological well-being variables, introducing the EEA trait resilience measures within a multiple regression in step 2 failed to produce a significant change in *R* ^2^ (subjective well-being, Δ*R* = .01, *p* = .924; psychological well-being, Δ*R* = .01, *p* = .518) from the significant regression models in step 1, predicted by the well-being at Time 1 (subjective well-being, *F* [1,99] = 45.63, *r* = .56, *r* ^2^ = .32, adj *r* ^2^ = .31, *p* < .001; psychological well-being, *F* [1,99] = 143.49, *r* = .59, *r* ^2^ = .61, adj *r* ^2^ = .59, *p* < .001). However, for physical health, introducing the EEA trait resilience measures in step 2 produced a significant change in *R* ^2^ (Δ*R* = .09, *p* = .006), adding to the significant regression models from step 1 (physical health, *F* [1,99] = 34.39, *r* = .51, *r* ^2^ = .26, adj *r* ^2^ = .25, *p* < .001). Engineering (B = -.72, β = -.20, *t* = -2.12, *p* = .036) and ecological (B = -1.10, β = -.24, *t* = -2.45, *p* = .016) trait resilience were found to predict unique variance in physical health at Time 2.

## Discussion

The findings from Study 1 suggest that a replicable three-factor model of trait resilience is viable, based on the three resilience systems described by Holling [e.g. 11, 13]. Based on a proposition derived by Kline (that the probity of latent trait constructs can be evidenced from the factor analysis of items), the current findings suggest that the EEA resilience factors emerge from the 115 items contained within five measures of trait resilience that are well-recognized in the psychological literature. From the EFA and CFA, it is possible to suggest a parsimonious 12-item EEA trait resilience measure that borrows items from these five existing psychological resilience measures, but uses a unique combination of items that cannot be formulated using a single resilience measure or using existing subscales from a single measure. What is worth noting about these proposed EEA items is that the item content reflects key dynamics that are emphasized in these resilience systems [13]. For example, the items that are used to measure engineering resilience do not just measure an ability to recover but also assess the swiftness of this recovery [13]. Similarly, the items that are used to measure the ecological resilience factor emphasize a person's ability to maintain their function whilst referring to processes that may accommodate or resist a disturbance [12]. Also, the items that are used to measure adaptive resilience reflect a person's willingness to both adapt and to vary their key functions. Study 2 presents data that show that the three EEA trait resilience scales demonstrate acceptable stability over a five-month period. This finding suggests that the new 12-item scale measures relatively stable resilience traits.

Study 2 considered the EEA trait resilience facets alongside wider trait psychology, and compared the three facets with models of personality and coping, while controlling for sex and age. The most prominent result from these comparisons is how different personality domains can be used to illustrate each of the EEA trait resilience facets, suggesting convergent validity for the EEA measure. Based on the criteria typically used to determine whether personality and coping predictors demonstrate standard beta coefficients of a moderate effect size, three findings emerge that are consistent with the general descriptions of the EEA trait resilience facets. First, engineering resilience is best understood within the low-neuroticism personality domain (emotional stability). Costa and McCrae [64] describe the emotionally stable individual as "calm, even-tempered and relaxed" (p. 15). This is consistent with the view that engineering resilience reflects the ability to return quickly to an equilibrium state [11, 13]. Equally, the facets of neuroticism comprise anger, hostility, depression, vulnerability, worry, and rumination, all of which are characteristics that may prevent individuals from returning to an equilibrium state. For example, depression, anxiety, worry, and rumination all comprise symptoms of distress that may be instrumental in an individual avoiding situations and behaviors that are important to recovering an equilibrium state [14, 64]. Second, ecological resilience is well-depicted by the conscientiousness personality domain. Costa and McCrae [64] described the conscientious individual as "purposeful, strong-willed and determined" (p. 16) and able to manage goals and maintain "active processes of planning, organizing and carrying out tasks" (p. 16) to reach goals. This is consistent with the view that ecological resilience reflects a system's ability to be robust, permanent, or persistent, to maintain a stable state (in terms of function, structure, or identity) that is generally resistant to perturbation, while simultaneously monitoring and reorganizing its structure or processes that govern its behavior in accommodating or resisting a disturbance [11, 13]. Third, adaptive resilience is well-described by the idea of openness to experience. Costa and McCrae [64] described the individual who is open to experience as curious and open to novel ideas, values, and experiences in relation to cognitions, behaviors, and affect. This is consistent with the view that adaptive resilience reflects the ability to vary one's key functions and processes [9, 12, 15–16].

It is worth noting one other personality factor that was also found to contribute unique variance to ecological and adaptive resilience in this study: extraversion. Although the reported effect sizes of the standardized beta coefficients are small, extraversion demonstrates a zero-order correlation with adaptive resilience that is larger than the zero-order correlation statistic found for openness to experience, and of a similar size (i.e. moderate) to the correlation with ecological resilience. Therefore, due to the probable variance in reported relationships between trait resilience and the five-factor model in future studies, it is necessary to record that extraversion (comprising warmth, excitement, activity, assertiveness, and excitement-seeking traits) may be a notable characteristic by which to describe and understand ecological and adaptive resilience. Notwithstanding likely variance in future studies, one key conclusion from Study 2 is that each facet of EEA trait resilience demonstrates a different substantial relationship with at least one of the key domains of the five-factor personality model. Additionally, the EEA resilience traits are significantly related to higher emotional stability, conscientiousness, openness to experience, and extraversion. These findings also support the prediction that the EEA resilience traits will fit within the general "adaptive landscape" of the five-factor model of personality [59, 60].

The proposition that EEA trait resilience reflects adaptive traits is further demonstrated by the relationship between the EEA trait resilience facets and well-being, with associations of small, moderate, and large effect sizes across different well-being dimensions and situations. Three elements are particularly striking. First, there is incremental value in two of the facets of the EEA model of trait resilience in predicting well-being after controlling for sex, age, personality, and coping. In this case, ecological resilience was able to predict both better subjective and psychological well-being and better self-reported health, while adaptive resilience is also able to predict better self-reported health. Second, engineering and ecological trait resilience are found to predict physical health over a five-month period, when controlling for levels of physical health at Time 1, thereby also suggesting that the EEA scales have predictive validity.

Some of these reported relationships between EEA trait resilience and well-being point to possible hypotheses through which future research might consider them. For example, we identified a close and predictive relationship between ecological resilience and psychological well-being. This suggests a "growth" hypothesis that ecological resilience, representing a system able to maintain its function and structure while simultaneously monitoring and reorganizing its processes, is aligned to eudemonic elements of existence and reflects a longer-term positive evaluation of purpose in life and engagement with life's challenges. Another possible hypothesis might be drawn from the findings that both engineering and ecological resilience are able to predict physical health over time. This suggests that systems that demonstrate resilience through the ability to return quickly to an equilibrium state (engineering) and the ability to maintain their function and structure while simultaneously monitoring and reorganizing their processes (ecological) are important to the maintenance of health systems. This perhaps suggests an "adjustment" hypothesis in which the EEA model of trait resilience might be useful for predicting changes in physical health over time.

The current findings suggest that the concept and measurement of EEA trait resilience may be useful for a number of areas of the psychological literature in which resilience is often applied—for example, health and well-being, education, and life-span [111–113]–not least because it is the first formulation of a trait resilience measure that directly assesses the EEA themes within the wider academic literature. However, further work is needed to consolidate some of the initial findings from the current studies. We suggest three limitations and further considerations to the current studies. First, it has to be recognized that the selection of the original items through the EFA was conducted among a sample in the United Kingdom and there were predominantly more women than men in the sample. Though this bias is typical of student populations studying psychological sciences in the United Kingdom, and though we found no gender-based differences for the EEA subscales among an older adult sample that was comprised of a more equal sex distribution, further research might consider the effects of gender-related differences on the scales. Likewise, the cross-cultural value of using this measure of EEA trait resilience should be further considered. Second, there are ways in which the proposed scales might be developed. Though a strength of the current study is the identification of EEA resilience as latent factors among current items used to measure resilience, given that the items were not directly created to measure EEA resilience constructs as described by Holling and colleagues, researchers might seek to examine whether improved items might be created that directly tap into the dynamics described in the EEA model. This would have the additional advantage of providing a new measure distinct from existing resilience measures that could then be used alongside these measures to examine other theories of resilience, such as positive appraisals [114]. Similarly, the proposed items currently place an emphasis on measuring resilience as a trait, while researchers often maintain that resilience is a process or state [29]. Therefore, future research might explore how the items could be used in a state context, measuring EEA resilience in response to specific situations, within specific time periods, and over time. Third, though the evidence from Study 2 clearly suggests that EEA trait resilience is related to a number of aspects of well-being, and can predict well-being over time, further research is needed to examine whether EEA trait resilience can be translated into intervention-type studies. This would be valuable for considering how the EEA trait resilience facets might be used to actively promote positive well-being outcomes.

## Conclusions

The studies herein present a refined conceptualization and method for considering trait resilience through three facets: engineering, ecological, and adaptive resilience as derived from a model of resilience described within ecological theory. The proposed items of the measure of EEA trait resilience have a pedigree, being derived from well-established measures of psychological resilience, and the three suggested dimensions demonstrate structural validity and stability over a five-month period. Moreover, EEA trait resilience shows both a relevance to, and a dynamic within, trait psychology through the facets’ various relationships with the dimensions of the five-factor model of personality and positive expressions of adaptive personality. Finally, the applied importance of EEA trait resilience is demonstrated by the facets’ association with well-being, in terms of their incremental value above models of personality and coping, and their longitudinal effects.

## Supporting Information

S1 Datasets Data used for the Exploratory Factor Analysis [Study 1] (Dataset A in S1 Datasets). Data used for the Confirmatory Factor Analysis [Study 1] (Dataset B in S1 Datasets). Data used for Personality, Coping, and Well-being Variables [Study 2] (Dataset C in S1 Datasets). Data used for the Test-Retest Study [Study 2] (Dataset D in S1 Datasets).
(ZIP)
